# Maternal overnutrition impairs offspring's insulin sensitivity: A systematic review and meta‐analysis

**DOI:** 10.1111/mcn.13031

**Published:** 2020-06-22

**Authors:** Szimonetta Eitmann, Dávid Németh, Péter Hegyi, Zsolt Szakács, András Garami, Márta Balaskó, Margit Solymár, Bálint Erőss, Enikő Kovács, Erika Pétervári

**Affiliations:** ^1^ Institute for Translational Medicine, Medical School University of Pécs Pécs Hungary; ^2^ János Szentágothai Research Centre University of Pécs Pécs Hungary; ^3^ Department of Thermophysiology, Institute for Translational Medicine, Medical School University of Pécs Pécs Hungary; ^4^ Division of Gastroenterology, First Department of Medicine, Medical School University of Pécs Pécs Hungary

**Keywords:** gestational weight gain, insulin resistance, insulin sensitivity, prepregnancy BMI

## Abstract

This systematic review and meta‐analysis aimed to investigate the association between maternal overnutrition and offspring's insulin sensitivity—following the Preferred Reporting Items for Systematic Reviews and Meta‐analyses statement. Studies published in English before April 22, 2019, were identified through searches of four medical databases. After selection, 15 studies aiming to explore the association between prepregnancy body mass index (ppBMI) or gestational weight gain (GWG) of non‐diabetic mothers and their offspring's insulin sensitivity (fasting insulin or glucose level and Homeostatic Measurement Assessment for Insulin Resistance [HOMA‐IR]) were included in the meta‐analysis. Associations of ppBMI and GWG with offspring's insulin sensitivity were analysed by pooling regression coefficients or standardized differences in means with 95% confidence intervals (CIs). Maternal ppBMI showed significant positive correlations with the level of both fasting insulin and HOMA‐IR in offspring (standardized regression coefficient for fasting insulin: 0.107, CI [0.053, 0.160], *p* < 0.001 and that for HOMA‐IR: 0.063, CI [0.006, 0.121], *p* = 0.031). However, the result of the analysis on coefficients adjusted for offspring's actual anthropometry (BMI and adiposity) was not significant. Independent from ppBMI, GWG tended to show a positive correlation with insulin level, but not after adjustment for offspring's anthropometry. Offspring of mothers with excessive GWG showed significantly higher HOMA‐IR than those of mothers with optimal GWG (*p* = 0.004). Our results demonstrate that both higher ppBMI and GWG increase the risk of offspring's insulin resistance, but the effect of ppBMI on insulin sensitivity in offspring may develop as consequence of their adiposity.

**Key messages**

- Evidence suggests that maternal overnutrition during pregnancy indicated by high BMI or GWG predisposes the growing fetus to metabolic disorders including obesity and insulin resistance leading to type 2 diabetes mellitus.
- Our meta‐analysis demonstrates an early‐onset positive linear association between ppBMI and parameters indicating insulin resistance in the offspring even without manifest hyperglycaemia, but this effect might be indirect via offspring's actual anthropometry (body weight and adiposity).
- Our meta‐analysis yields a suggestive, but still limited statistical evidence for a positive association of excessive GWG with offspring's insulin resistance.

## 1 INTRODUCTION

Obesity is a global health hazard, and its frequency in adults and even in reproductive‐aged women is growing dramatically (World Health Organization [WHO], 2018). Obesity affects 20%–38% of all pregnancies and increases the risk of obesity and metabolic diseases in both children and adult offspring (Tenenbaum‐Gavish & Hod, 2013). Based on the US Institute of Medicine (IOM) guidelines, about 40% of women gain an excessive amount of weight during pregnancy in Western countries (IOM, 2009). The IOM guidelines define optimal ranges of gestational weight gain (GWG) during pregnancy according to a mother's prepregnancy body mass index (ppBMI). Increasing evidence suggests that adult‐onset metabolic disorders may derive in part from events taking place during fetal and early postnatal development (Iozzo et al., 2014). A large body of evidence suggests that maternal obesity is accountable for the direct transmission of obesogenic and diabetogenic phenotypes to the succeeding generation. According to the concept of Developmental Origin of Health and Disease, maternal obesity and accelerated growth in neonates predispose offspring to obesity and cardiometabolic diseases even in adulthood (Agarwal et al., 2018). Developmental programming refers to the ability of factors during prenatal and neonatal life to cause long‐term effects in adults. Intrauterine environment (e.g., maternal overnutrition) may influence fetal growth by metabolic developmental programming and thus leads to lifelong physiological changes that predispose the body to metabolic diseases, for example, metabolic syndrome. Metabolic syndrome (also called insulin resistance syndrome) that includes abdominal obesity, insulin resistance (IR) or type 2 diabetes mellitus (T2DM), systemic hypertension and atherogenic dyslipidaemia is associated with a higher risk for cardiovascular mortality (McCracken, Monaghan, & Sreenivasan, 2018). Decreased insulin sensitivity (IS) is believed to be a critical pathophysiological event early in the disease process (Thompson & Regnault, 2011). Most observational studies confirmed the link between high GWG or BMI (used as indicators of a high‐calorie nutritional environment during pregnancy, Symonds, Sebert, Hyatt, & Budge, 2009) and offspring's obesity (Drake & Reynolds, 2010; Heslehurst et al., 2019; Mamun, Mannan, & Doi, 2014). The impact of obesity on IR is well‐known even in children (Thota, Perez‐Lopez, Benites‐Zapata, Pasupuleti, & Hernandez, 2017). However, the data about the association between obesity of non‐diabetic mothers and offspring's IR are controversial (Gaillard et al., 2016; Hochner et al., 2012; Jeffery et al., 2006; Tam et al., 2018). To date, no systematic review has analysed the impact of maternal overnutrition on offspring's IS. It is still not clarified, whether this effect is only indirect (offspring's obesity enhances the risk of IR) or the intrauterine environment acts also directly on IS. We, therefore, aimed to review the literature complemented by a meta‐analysis to investigate the impact of the following factors on offspring's IS: (1) high ppBMI and (2) excessive GWG independently from ppBMI. We also aimed to analyse the potential influence of offspring's actual BMI or body weight on these associations. We hypothesized positive correlations of both ppBMI and GWG with offspring's parameters characterizing IR and that these associations are mediated by childhood adiposity.

## 2 METHODS

### 2.1 Search strategy

The review was conducted by searching MEDLINE (via PubMed), EMBASE, Scopus and CENTRAL databases until April 22, 2019 (search strategy: Appendix A). The protocol was registered onto the International Prospective Register of Systematic Reviews (PROSPERO).

### 2.2 Study selection

Using Preferred Reporting Items for Systematic Reviews and Meta‐analyses (PRISMA, checklist: Appendix B) (Shamseer et al., 2015), two researchers (S. E. and E. K.) conducted the screening and data extraction independently. After screening for title and abstract, all potentially relevant studies were retrieved for full‐text evaluation. Disagreements were resolved by a third reviewer (E. P.). To be included, each article had to provide data about maternal overnutrition just before or during pregnancy in healthy mothers along with data about their offspring's IS. Further inclusion criteria were either exclusion of pathological (e.g., preeclampsia and gestational DM) and twin pregnancies from the study or presence of adjustment for these conditions. We did not use restriction on offspring's age or study design. The exclusion criteria were animal experiments, non‐English studies and studies providing maternal data only after birth. If duplicate studies were found within the same data source, the larger population was selected. We extracted the following data from each study: first author, year of publication, country, study setting with year of enrolment or data collection, inclusion and exclusion criteria, maternal age at delivery, timing of the maternal measurement, number of participants, offspring's age and gender, maternal nutritional status just before or during pregnancy, data about offspring's IS, data describing the association between the maternal data and their offspring's IS and adjusted covariates. In case of a linear type of association, the regression or correlation coefficient was recorded. In case of categorization based on the maternal nutritional state the mean difference, the odds ratio or risk ratio was recorded. The values adjusted for most confounders were extracted.

### 2.3 Risk of bias assessment

Two independent investigators (S. E. and E. K.) performed the quality assessment separately, and disagreements were resolved by a third author (E. P.). A critical appraisal tool, the Quality in Prognosis Studies (QUIPS) was used to assess the methodological quality of the identified studies (Hayden, van der Windt, Cartwright, Cote, & Bombardier, 2013). QUIPS covers six main domains: ‘Study participation’, ‘Study attrition’, ‘Prognostic factor’, ‘Outcome measurement’, ‘Study confounding’ and ‘Statistical analysis and reporting’. For each item of the domains, ‘yes’, ‘no’, ‘partly’ or ‘unclear’ was used to assess the risk of bias. An overall rating for each domain was assigned as carrying ‘low’, ‘moderate’ or ‘high’ risk of bias. Moreover, due to weighting methods, data with low participant numbers were assigned with lower weights during the analysis.

### 2.4 Data analysis

We used random effect models with the DerSimonian and Laird weighting method in meta‐analysis to give a summary point estimate along with a 95% confidence interval (CI) and *p* < 0.05 was regarded as statistically significant. The relative weights of the individual studies were calculated using the number of included studies, and the individual study‐specific estimates and standard errors (depending on CI and number of participants) of each included study (Borenstein, Hedges, Higgins, & Rothstein, 2010). With regard to the association between maternal nutritional state and offspring's parameters, we analysed either regression coefficients or standardized differences in means (SMD) of offspring's parameters in GWG categories.

Because the regression coefficient can be provided in standardized (beta) or unstandardized forms (*B*), if it was available, we collected the corresponding standard deviations to convert *B* or beta coefficients to each other. For this computation, we used the following equation: beta = (B*SD_*x*_)/SD_*y*_, where SD_*x*_ and SD_*y*_ are the standard deviations of the independent variable (maternal data) and the dependent variable (offspring's data), respectively (Vittinghoff, Glidden, Shiboski, & McCulloch, 2005). Correlation coefficients (*r*) were used to compute beta with the following equation: beta = (r*SD_*y*_)/SD_*x*_ (Kenney & Keeping, 1962).

To test if the association is an at least partly direct effect of maternal overnutrition on offspring's IS or it is only indirect via offspring's actual anthropometric characteristics (body weight, BMI and adiposity), we used two models: we pooled regression coefficients (1) adjusted for offspring's anthropometry and (2) without this adjustment. We could carry out analyses if data from at least three studies were available per association. The available data allowed sensitivity analysis for four major confounders: we performed the tests without those studies which were not adjusted for maternal age, smoking, offspring's gender and birth weight (BW).

To assess statistical heterogeneity, *Q* test and I‐squared statistics were calculated. We considered the *Q* test significant if *p* < 0.1. I‐squared statistics represents the percentage of effect size heterogeneity that cannot be explained by random chance. Heterogeneity could be interpreted as moderate between 30% and 60%, as substantial between 50% and 90% and as considerable above 75% (The Cochrane Collaboration, 2011).

To test the presence of publication bias (small‐study effect) we assessed the symmetry of the funnel plots visually.

All statistical analyses were performed with Comprehensive Meta‐analysis Software Version 3 (Biostat, Inc., USA).

## 3 RESULTS

### 3.1 Search, selection and characteristics of the studies

**FIGURE 1:**
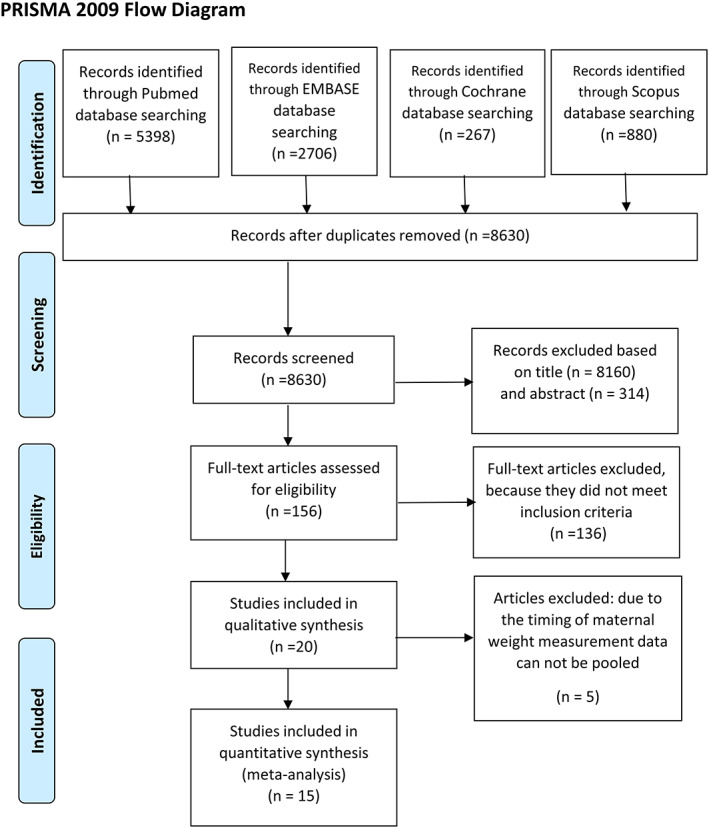
Flowchart of the study selection process

The systematic search produced 9,251 records. After removing duplicates, screening of titles, abstracts and full‐text papers for eligibility, 20 observational studies were included in the qualitative synthesis (Figure 1), 15 of which were eligible for meta‐analysis (Brandt et al., 2014; Dello Russo et al., 2013; Derraik, Ayyavoo, Hofman, Biggs, & Cutfield, 2015; Gaillard, Steegers, Franco, Hofman, & Jaddoe, 2014; Gaillard et al., 2016; Hochner et al., 2012; Hrolfsdottir et al., 2015; Jeffery et al., 2006; Maftei et al., 2015; Mingrone et al., 2008; Oostvogels et al., 2014; Perng, Gillman, Mantzoros, & Oken, 2014; Sauder, Hockett, Ringham, Glueck, & Dabelea, 2017; Tam et al., 2018; Winham, Johnston, & Rhoda, 2006: Table 1). The remaining five studies were also eligible for the inclusion, but their data could not be statistically pooled due to the timing of maternal measurement: BMI was measured at different times of the third trimester (Bucci et al., 2016; Eriksson, Sandboge, Salonen, Kajantie, & Osmond, 2014; Mi et al., 2000; Shaikh, Basit, Hakeem, Fawwad, & Hussain, 2015; Veena, Krishnaveni, Karat, Osmond, & Fall, 2013). Only one of them (Veena et al., 2013) provided the sum of maternal skinfold thickness instead of weight measurement. Characteristics of these studies are shown in Appendix C.

**TABLE 1:** Characteristics of the included studies

Author, year, country	Study design	Maternal characteristics				Offspring's characteristics	
		Age^a^ (year)	Sample size	ppBMI assessment	GWG assessment	Age^a^ (year)	Male (%)
Brandt et al., 2014, Germany	Prospective	32.7 (32.4–33.0)	249	Records	Total GWG: Records	8	49.3
Dello Russo et al., 2013, Germany, Hungary, Italy, Cyprus, Spain, Estonia, Sweden, Belgium	Retrospective	28.4 (28.2–28.6)	2,180		Total GWG: Self‐reported	2–9	52.8
Derraik et al., 2015, New Zealand	Retrospective	17–42	70	Self‐reported weight		4–11	61
Gaillard et al., 2014, The Netherlands	Prospective	30.3 ± 5.1	3,877 (GWG in unadjusted model: 2640)	Self‐reported/measured weight	Maximum GWG: Self‐reported/measured	5.6–8	49.9
Gaillard et al., 2016, Australia	Prospective	29 ± 5.8	1,392	Self‐reported weight	Total GWG: Measured	16.7–17.7	50.7
Hochner et al., 2012, Israel	Prospective	28.4 ± 5.5	1,134	Self‐reported weight	Maximum GWG: Records	32 ± 0	49.5
Hrolfsdottir et al., 2015, Denmark	Prospective	29.0 ± 4.9	308		Maximum GWG: Records	22.3 ± 3.0	39.3
Jeffery et al., 2006, United Kingdom	Prospective	34	230	Self‐reported weight		8 ± 0	53.3
Maftei et al., 2015, Australia	Prospective	30.3 ± 5.1	163	Self‐reported weight	Total GWG: Records	9–10	45
Mingrone et al., 2008, Italy	Retrospective	21–29	52	Self‐reported weight		23.8 ± 4.5	40
Oostvogels et al., 2014, The Netherlands	Prospective	31.1 ± 4.7	1,459	Self‐reported weight		5–6	53
Perng et al., 2014, USA	Prospective	15–44	677 (adjusted model) 592 (unadjusted model)	Self‐reported weight	Total GWG: Records	7.7 (6.6–10.9)	49.7
Sauder et al., 2017, USA	Prospective	30.0 ± 5.4	236	Records		16.6 ± 1.2	51
Tam et al., 2018, China	Prospective	31.3 ± 4.6	371		Total GWG: Records	7 ± 0	52
Winham et al., 2006, USA	Retrospective	21.8 ± 1.7	8		Total GWG: Records	0.5 ± 0	56

Abbreviations: GWG, gestational weight gain; ppBMI, prepregnancy BMI.

^a^ Age is expressed in mean ± standard deviation or range or median (interquartile range).

The 15 studies which could be included in the quantitative analysis contained statistically sufficient data and described maternal overnutrition as ppBMI (assessed just before pregnancy or at the first antenatal visit) or GWG (total or maximum). Twelve of which reported maternal and offspring's data as continuous variables and provided *B*, beta or *r* coefficients for describing their link. Three articles provided data on offspring's IS by GWG categories in accordance with the 2009 IOM guidelines (Dello Russo et al., 2013; Hrolfsdottir et al., 2015; Tam et al., 2018).

In the majority of the studies, prepregnancy body weight was self‐reported for the assessment of ppBMI. GWG was computed across most of the studies as the difference between the last measured weight at the end of pregnancy and either prepregnancy weight or the first prenatal visit weight. Three studies, however, derived GWG from the maximum weight at pregnancy rather than the last weight at pregnancy (Gaillard et al., 2014; Hochner et al., 2012; Hrolfsdottir et al., 2015). For the estimation of offspring's IS serum level of fasting insulin (nine articles) or fasting glucose (seven articles) and Homeostatic Measurement Assessment for Insulin Resistance (HOMA‐IR, estimated from the fasting insulin‐glucose product, divided by 22.5; eight articles) were used applying the standardized laboratory methods. Other parameters were not convertible to a common unit, or the number of studies reporting other parameters was not sufficient for statistics (C peptide: one article, Gaillard et al., 2014; acute insulin response in glucose tolerance tests: two articles, Derraik et al., 2015; Mingrone et al., 2008).

The included 15 articles were published from 2006 to 2018 and provided data of 12,406 mother–offspring pairs from 16 countries. The number of participants (mother–offspring pairs) ranged from 8 to 3,877 per study. Caucasians were the dominant ethnic group as the studies were mostly from Europe and the United States. Eleven articles were prospective studies; only four articles were retrospective cohort studies. All included studies reported women who had singleton full‐term pregnancies with a maternal age ranging between 15 and 44 years (at childbirth). The offspring's age at outcome assessment ranged 0.5–32 years; three studies focused on adults (19–32 years) (Hochner et al., 2012; Hrolfsdottir et al., 2015; Mingrone et al., 2008). All studies contained pooled data of males and females.

Most of the studies examined confounders (Appendix D). Commonly considered variables were maternal confounders (e.g., age, smoking during pregnancy, socioeconomic status [SES: educational level and income], gestational age and breastfeeding) and offspring's covariates (e.g., BW, age and gender). Coefficients adjusted for offspring's actual anthropometry (BMI, total fat mass index and weight‐for‐length gain) in seven studies allowed the separate analysis of this variable as a potential mediating factor.

### 3.2 Study quality

The detailed results of the risk of bias assessment according to the adapted QUIPS tool can be found in Appendix E. The main domain ‘Study attrition’ was not suitable for the retrospective studies. ‘Study participation’, ‘Study attrition’ and ‘Statistical analysis and reporting’ domains were evaluated as low or moderate risk of bias in all studies. The domains ‘Prognostic factor measurement’ (i.e., maternal nutritional state) and ‘Outcome measurement’ (i.e., offspring's parameters) were the best rated: all studies were judged to carry a low risk of bias. In contrast, 60% of the studies showed a high risk of bias considering the domain ‘Study confounding’, because they did not report how the important confounders were accounted for in the analysis. Seven of these latter studies had the highest risk of bias because they were rated as high risk of bias in one domain (‘Study confounding’) and additionally as moderate risk in one other domain (Brandt et al., 2014; Derraik et al., 2015; Hrolfsdottir et al., 2015; Maftei et al., 2015; Sauder et al., 2017; Tam et al., 2018; Winham et al., 2006). The four studies that had the lowest risk of bias were rated in only one domain as moderate and in all other domains as low risk (Gaillard et al., 2014; Gaillard et al., 2016; Hochner et al., 2012; Oostvogels et al., 2014).

### 3.3 Linear type of association between ppBMI and offspring's IR

**FIGURE 2:**
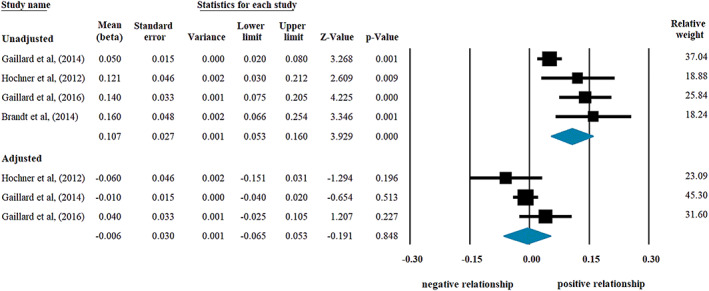
Beta regression coefficients describing the association between prepregnancy body mass index (BMI) and offspring's insulin level without (upper panel; heterogeneity: *I* ^2^ = 71.81%, *p* = 0.014) or with adjustment for offspring's BMI (lower panel; *I* ^2^ = 40.32%, *p* = 0.187). Black squares show beta values with the area reflecting the weight assigned to the individual studies. Horizontal bars indicate 95% confidence intervals. Diamonds show the overall point estimate with 95% confidence intervals

With regard to studies that investigated the association between ppBMI and offspring's insulin level, the random effect model showed a significant positive correlation (beta regression coefficient: 0.107, CI [+0.053, +0.160], *p* < 0.001; Figure 2, upper panel). Even without the study (Brandt et al., 2014) in which beta was not adjusted for maternal age, smoking, SES and offspring's BW, the association remained significant (beta: 0.094, CI [+0.038, 0.150], *p* = 0.001). In contrast, analysis of coefficients adjusted for offspring's actual BMI failed to show any effect of ppBMI (beta: −0.006, CI [−0.065, +0.053], *p* = 0.848; Figure 2, lower panel). Similar results were found if unstandardized *B* regression coefficients were used (reflecting the increase in fasting insulin level given in pmol/L with every 1 kg/m^2^ increase in ppBMI). The increase was also significant in the model without adjustment for offspring's BMI: 0.010, CI [+0.003, +0.016], *p* = 0.004 (Brandt et al., 2014; Derraik et al., 2015; Gaillard et al., 2014; Gaillard et al., 2016; Hochner et al., 2012; Mingrone et al., 2008). In the analysis of *B* adjusted for offspring's BMI a lack of association (*p* = 0.765) was found (Gaillard et al., 2014; Gaillard et al., 2016; Hochner et al., 2012).

In contrast, analyses in models applying coefficients unadjusted versus adjusted for offspring's BW did not show different results. Similarly significant results were obtained from the analysis of beta coefficients unadjusted for BW and actual BMI (Appendix F; *p* = 0.004) and from that of coefficients adjusted for BW but not for actual BMI (above the sensitivity analysis without the study of Brandt et al.).

**FIGURE 3:**
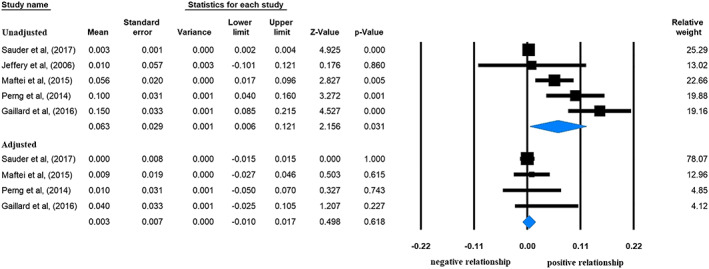
Beta regression coefficients describing the association between prepregnancy body mass index and offspring's Homeostatic Measurement Assessment for Insulin Resistance (HOMA‐IR) without (upper panel; heterogeneity: *I* ^2^ = 89.15%, *p* < 0.001) or with adjustment for offspring's anthropometry (lower panel; *I* ^2^ = 93.27%, *p* < 0.001). Black squares show beta values with the area reflecting the weight assigned to the individual studies. Horizontal bars indicate 95% confidence intervals. Diamonds show the overall point estimate with 95% confidence intervals

With regard to those studies that investigated the association between ppBMI and offspring's HOMA‐IR values, the analysis also showed a significant positive correlation (beta: 0.063, CI [+0.006, +0.121], *p* = 0.031; Figure 3, upper panel). Here, the offspring's age was under 18 years, and these children were healthy with still normal glucose levels. Therefore, this result indicates an early‐onset positive linear association between ppBMI and HOMA‐IR indicating IR in children without manifest hyperglycaemia. Even without the studies (Jeffery et al., 2006; Maftei et al., 2015) in which beta was not adjusted for maternal age, smoking and offspring's gender, the association remained significant (0.090, CI [+0.016, +0.164], *p* = 0.018). However, coefficients from two studies (Perng et al., 2014; Sauder et al., 2017) were not adjusted for maternal SES and offspring's BW; these results could be biased by these covariates. The analysis of coefficients adjusted for offspring's actual anthropometry showed lack of association (beta: 0.003, CI [−0.010, +0.017], *p* = 0.618; Figure 3, lower panel). In the case of these latter associations and also in the following tests, we could analyse only beta instead of the *B* coefficients due to the lack of data.

In contrast, we did not find any association between ppBMI and offspring's glucose level. Beta was non‐significant both without adjustment for offspring's anthropometry (0.004, CI [−0.008, +0.017], *p* = 0.500; *I* ^2^ = 64.38%, *p* = 0.060) and with this adjustment (0.002, CI [−0.010, +0.015], *p* = 0.713; *I* ^2^ = 1.66%, *p* = 0.362; Gaillard et al., 2016; Hochner et al., 2012; Oostvogels et al., 2014).

### 3.4 Linear type of association between GWG and offspring's IR

**FIGURE 4:**
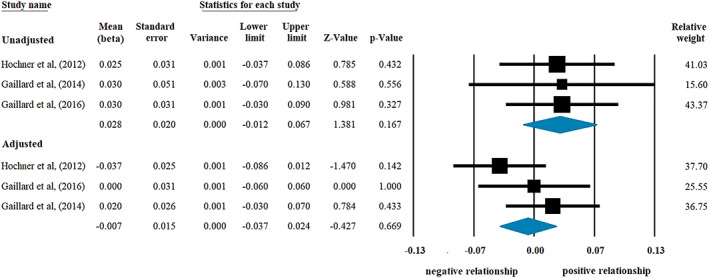
Beta regression coefficients describing the association between gestational weight gain and offspring's insulin level without (upper panel; heterogeneity: *I* ^2^ = 0%, *p* = 0.992) or with adjustment for offspring's body mass index (lower panel; *I* ^2^ = 22.85%, *p* = 0.274). Black squares show beta values with the area reflecting the weight assigned to the individual studies. Horizontal bars indicate 95% confidence intervals. Diamonds show the overall point estimate with 95% confidence intervals

A tendency for a positive association was detected in the relationship between GWG and offspring's insulin level, but the result did not reach statistical significance (beta: 0.028, CI [−0.012, +0.067], *p* = 0.167; Figure 4, upper panel). The same result was found if we included the study (Winham et al., 2006) in which beta was not adjusted for maternal age, smoking, SES, offspring's BW and gender (beta: 0.075, CI [−0.009, +0.159], *p* = 0.080; *I* ^2^ = 86.27%, *p* < 0.001; *n* = 4). Analysis of offspring's BMI as a covariate in the statistical model showed a lack of association (−0.007, CI [−0.037, +0.024], *p* = 0.669; Figure 4, lower panel).

With regard to offspring's HOMA‐IR values, similar results were obtained. Beta was non‐significant both without adjustment for offspring's anthropometry (0.009, CI [−0.020, +0.038], *p* = 0.549; *I* ^2^ = 0%, *p* = 0.701; *n* = 3) and with this adjustment (−0.018, CI [−0.044, +0.008], *p* = 0.173; *I* ^2^ = 0%, *p* = 0.804; Gaillard et al., 2016; Maftei et al., 2015; Perng et al., 2014). Maternal smoking, age, SES, offspring's BW and gender could not be taken into consideration in these analyses.

### 3.5 Association between GWG (according to IOM categories) and offspring's IR

**FIGURE 5:**
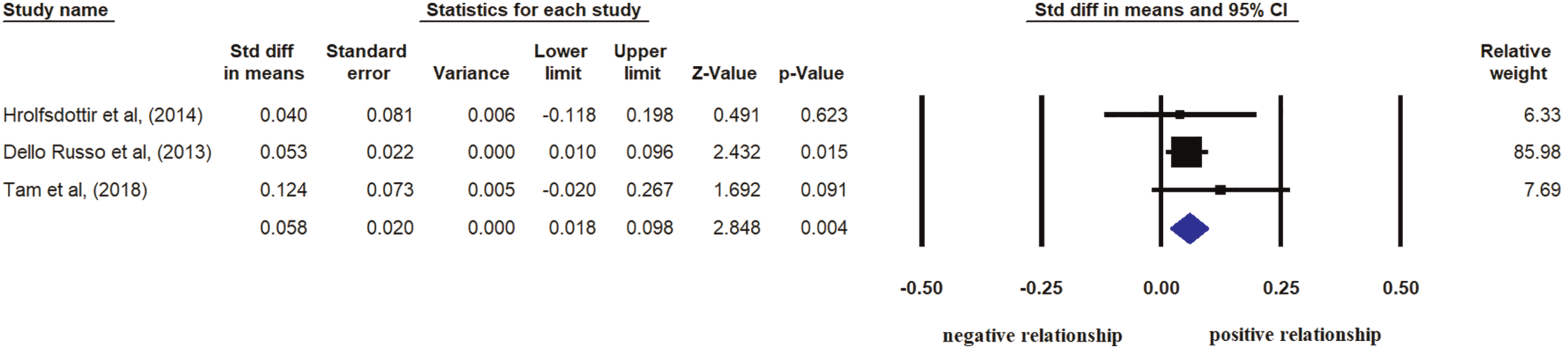
Standardized differences in mean Homeostatic Measurement Assessment for Insulin Resistance (HOMA‐IR) values in offspring of mothers with excessive gestational weight gain (GWG) compared with those of adequate GWG. Black squares show the differences in mean values with the area reflecting the weight assigned to the individual studies. Horizontal bars indicate 95% confidence intervals. The diamond shows the overall point estimate with 95% confidence interval (heterogeneity: *I* ^2^ = 0%, *p* = 0.635)

Three articles provided offspring's fasting insulin level and HOMA‐IR values as outcomes in the three GWG categories (i.e., suboptimal, adequate and excessive); therefore, we could compare the differences in mean HOMA‐IR values or insulin levels in offspring of mothers with excessive versus adequate GWG (Dello Russo et al., 2013; Hrolfsdottir et al., 2015; Tam et al., 2018). In contrast to the analyses of linear models which included offspring of mothers with suboptimal GWG, application of categorization‐based analysis yielded significant results: higher mean HOMA‐IR values in offspring of mothers who had excessive GWG than those of adequate GWG group (SMD: 0.058, CI [+0.018, +0.098], *p* = 0.004; Figure 5). Concerning insulin level, the difference did not reach statistical significance (0.076, CI [−0.040, +0.193], *p* = 0.198; *I* ^2^ = 68.52%, *p* = 0.042).

Comparison of parameters in offspring of mothers of suboptimal versus adequate GWG groups failed to show any significant difference (SMD for HOMA‐IR: 0.000, CI [−0.122, +0.121], *p* = 0.994; *I* ^2^ = 64.21%, *p* = 0.061, for insulin: −0.029, CI [−0.117, +0.059], *p* = 0.517; *I* ^2^ = 40.32%, *p* = 0.187).

All data in these categorization‐based analyses were adjusted for maternal age, offspring's age and gender. Maternal smoking, SES, offspring's BW and actual anthropometry could not be taken into consideration as covariates, but all participants had BW in normal range. Data of only one study were not adjusted for maternal smoking (Tam et al., 2018), but the negligible percentage of smoking mothers (4 from 2,859 mothers) was unlikely to influence the results.

### 3.6 Publication bias

Visual inspection of the funnel plots suggested no small‐study effect. Only one study (Winham et al., 2006) could have an excessive influence on the pooled effect size, but the analysis with or without this study showed similar results (Figure 4). Thus, our results appear not to be influenced by small‐study effect.

## 4 DISCUSSION

Maternal obesity during pregnancy is associated with an unfavourable environment for the growing fetus and predisposes offspring to obesity, IR and T2DM (Catalano, Presley, Minium, & Hauguel‐de Mouzon, 2009; Eriksson et al., 2014; Mingrone et al., 2008). The changes of the intrauterine programming due to maternal obesity interacts with offspring's genetic characteristics and seems to be more important than genetic factors to determine adult life health both in humans (Vaag et al., 2014) and in animal models with low genetic vvariability (Gluckman, Hanson, Cooper, & Thornburg, 2008). Furthermore, there is a stronger correlation between maternal BMI and adiposity in children compared with paternal BMI. Thus, besides genetic factors and the shared family‐based, lifestyle‐related characteristics, the intrauterine environment may also significantly contribute to the development of offspring's obesity (Danielzik, Langnase, Mast, Spethmann, & Muller, 2002). Both ppBMI and GWG are proxies for early nutritional environment and have significant association with offspring's BMI. The strength of the effect of GWG is generally weaker than that of maternal obesity per se, but it seems to be stronger among underweight/normal‐weight women. This highlights the importance of avoiding excessive GWG even in underweight/normal‐weight women (Mamun et al., 2009). However, there are contradictory findings concerning the impact of maternal overnutrition on the IR with or without obesity. IR tracks from early life periods into adulthood (Thompson & Regnault, 2011) leading to T2DM and a global disease burden (WHO, 2018). Thus, the evaluation of early life factors contributing to IR is highly relevant.

Our meta‐analysis demonstrated a positive linear association between ppBMI and offspring's IR indicated by increases in fasting insulin level and HOMA‐IR. Our meta‐analysis yielded statistical evidence for higher HOMA‐IR associated with excessive GWG (independent of ppBMI). The association of ppBMI with insulin level is independent of maternal age, smoking, SES, offspring's age, gender and BW, because in our analysis, the coefficients were adjusted for all these important covariates. However, the lack of associations of ppBMI with offspring's insulin level and HOMA‐IR in the model adjusted for offspring's actual anthropometry indicates that IR does not develop independent of offspring's adiposity, just as its consequence. This finding suggests that the relationship between ppBMI and offspring's IR is indirect; the development of IR might be mediated via offspring's adiposity. Therefore, reduction of obesity in childhood could help prevent the development of IR and complications arising from early life exposures. Investigation of underlying mechanisms in animal experiments revealed that in fetuses of overnourished sheep, increased fatty acid transport from maternal circulation induces inflammation which contributes to a shift from myogenesis to adipogenesis indicated by enhanced expression of peroxisome proliferator‐activated receptor gamma in skeletal muscles (Zhu et al., 2008; Zhu, Ma, Long, Du, & Ford, 2010). Increased tumour necrosis factor‐alpha as indicator of inflammation has been shown to reduce activation of AMP‐activated protein kinase (AMPK), which could result in progressive lipid accumulation. Intramuscular fat accumulation with significant reduction of AMPK activity induced intrauterine functional impairment of insulin signalling (Yan et al., 2010; Zhu et al., 2008). These impairments could lead to postnatal adiposity indicated by higher body weight and then later appearance of decreased IS in mouse offspring exposed to high‐fat diet in utero (Masuyama & Hiramatsu, 2012).

With regard to glucose level (considering all covariates), we did not find significant association. The available studies included children and young adults; therefore, our results suggest an early‐onset IR before any detectable increase in fasting glucose level within the normal range and before the manifestation of hyperglycaemia or T2DM. According to earlier observations, an excessive increase in BMI even after the age of 2 years predicts the development of IR in later life (Barker, 2005).

Although IS was not measured in the available studies using the hyperinsulinaemic‐euglycaemic clamp technique, HOMA‐IR is considered a valid alternative to estimate IS and has been shown to predict imminent T2DM (Ghasemi et al., 2015). As a confirmation of our results, two sets of data from included studies (that we could not include in our statistical analysis) demonstrated higher glucose level or worse insulin response to oral or intravenous glucose administration in offspring of obese/overweight mothers, respectively (Derraik et al., 2015; Mingrone et al., 2008). In accordance with our results, a recent study demonstrated decreased muscle IS even in 70–73 years old daughters of obese/overweight mothers as compared with offspring of lean/normal mothers (Bucci et al., 2016). When the comparison was corrected for offspring's BMI, the significance was lost. This finding also suggests that the association could be explained by offspring's anthropometry. Accordingly, lean young adult (20–26 years old) offspring of obese parents failed to show lower IS compared with offspring of normal‐weight parents (Lazarin et al., 2004). Our findings are in agreement with recent reports that described similar positive relationships between maternal overweight/obesity and either T2DM in adult offspring (Eriksson et al., 2014) or metabolic syndrome in children (González‐Jiménez, Montero‐Alonso, Schmidt‐RioValle, García‐García, & Padez, 2015) or even in young adults (Delpierre et al., 2016). These studies underline the importance of our findings concerning early appearance of IR in offspring of obese/overweight mothers.

Obesity in pregnancy is commonly associated with heightened risk for gestational diabetes. Our meta‐analysis focused on non‐diabetic, singleton pregnancies. In contrast to our results, exposure to maternal diabetes has been shown to be associated with higher IR in children, independent of both maternal ppBMI and offspring's BMI (Lowe et al., 2019; Sauder et al., 2017). Contrary to singletons, twins' IR fell as their mothers' ppBMI increased (Loos et al., 2002).

With regard to GWG independent from ppBMI (considering all important covariates), our meta‐analysis tended to show a non‐significant positive correlation with insulin level, but not in model adjusted for offspring's actual anthropometry. The lack of significant result (i.e., lack of linear association) may suggest a non‐linear association of GWG (on continuous scale from suboptimal to excessive) with offspring's IR. In our analysis, GWG on a continuous scale included not only mothers with excessive and adequate but also those with suboptimal weight gain according to IOM categories. Earlier observations showed higher insulin levels in offspring of undernourished mothers (Mi et al., 2000). Therefore, instead of a linear type of association, a U‐shaped relationship was suggested in a recent study: the risk for IR increased to both the lower and upper extremes of GWG (Tam et al., 2018). If we compared IR in offspring of mothers with excessive versus adequate GWG (without suboptimal category), we found significantly higher HOMA‐IR in the excessive GWG‐group independent of maternal age, smoking, offspring's BW, age and gender. However, we could not confirm the U‐shaped relationship to the lower extremes of GWG suggested recently by Tam et al., (2018). Further studies are needed to clarify the relationship in the lower extremes of GWG.

The link between maternal and child obesity may partially track through BW. Maternal weight (or BMI) seems to be a more important risk factor for obesity in the child than BW, because it could partially explain the association between BW and offspring's BMI (Parsons, Power, & Manor, 2001). However, it is known that BW depends not only on maternal overnutrition but it reflects other factors (e.g., in utero hypoxia and birth defects) potentially influencing the development of IR. Thus, BW is also on the pathway between ppBMI and IS in the same way as offspring's BMI. Offspring in both high and low BW categories showed a greater risk of T2DM compared with normal BW (Knop et al., 2018). However, in our meta‐analysis, we demonstrated significant positive association between ppBMI and offspring's insulin level independent of BW, but dependent of offspring's actual anthropometry. We also demonstrated significantly higher HOMA‐IR in normal BW offspring of mothers with excessive GWG than those with adequate GWG.

Maternal age at childbirth represents an important confounder, and U‐shaped associations have been described with offspring's fasting glucose and T2DM (Fall et al., 2015; Lammi et al., 2007). Sons of both younger and older mothers had higher HOMA‐IR values than those of mothers aged 30–34; these associations were only partly dependent on BW, pregnancy complications and maternal educational level as a proxy for SES. Programming effects of hormonal imbalances related to maternal age and epigenetic modifications are important mechanisms underlying the association of maternal age with offspring's metabolic parameters (Verroken, Zmierczak, Goemaere, Kaufman, & Lapauw, 2017). In addition, both low maternal educational level and low economic status have been shown to be associated with higher ppBMI, excessive GWG (Huynh, Borrell, & Chambers, 2014; Park et al., 2011) and higher offspring's glucose level and HOMA‐IR (van den Berg, van Eijsden, Vrijkotte, & Gemke, 2012). The association with glucose profile was dependent on childhood BMI (van den Berg et al., 2012), similar to our observation with regard to the relationship between ppBMI and offspring's insulin level. These findings suggest the possibility that low maternal education could influence offspring's IR indirectly via maternal overnutrition. Maternal smoking during pregnancy has also been shown to increase the risk for T2DM in offspring, but this association was largely explained by offspring's BMI (Jaddoe et al., 2014). Possible mechanisms include toxins in tobacco smoke, impaired uteroplacental blood flow leading to smaller BW (Rogers, 2019). In our meta‐analysis, maternal age and smoking did not influence our conclusions concerning significant associations between ppBMI and offspring's insulin level or HOMA‐IR. Similarly, these confounders did not bias the significant result on the relationship between excessive GWG and HOMA‐IR. However, these results on the link with HOMA‐IR might be biased by maternal SES. Significant contribution of other confounders (e.g., breastfeeding) is still not clarified; they could limit the results of our meta‐analysis (Delpierre et al., 2016; González‐Jiménez et al., 2015). Because the impact of the mentioned maternal factors on offspring's later health is obvious, and maternal smoking, overnutrition and age at childbirth are increasing worldwide, effective behavioural interventions are needed to address obesity in women.

The investigated parameters of IR do not depend on age or gender (Reinehr, 2013). Nevertheless, an earlier study described higher insulin secretion in adult sons of obese mothers than in daughters, but their IS did not differ (Mingrone et al., 2008). All studies involved in our conclusive results applied adjustment for offspring's age and gender; thus, our findings could not be biased by this covariates.

### 4.1 Strengths and limitations

One of the main strengths of our paper is that our meta‐analysis is the first to investigate the impact of ppBMI and GWG separately on offspring's IS. Moreover, further strengths include the high number of participants and exclusion of pathological and twin pregnancies. In pathological pregnancies, other mechanisms may lead to a higher weight in pregnancy and diseases in offspring (Boney, Verma, Tucker, & Vohr, 2005). In addition, we separately investigated the direct versus indirect associations concerning offspring's actual anthropometry. Thorough analysis of the potential influence of the most important maternal and offspring's covariates (maternal age, smoking, offspring's gender and BW) showed reliability of conclusive results.

Limitations include the lack of investigation of gender differences, specific confounders (e.g., SES), self‐reported prepregnancy weight in most of the studies. However, self‐reported and measured maternal weight showed strong correlation in earlier studies (Mamun et al., 2011; Thomas, Paulet, & Rajpura, 2016). With regard to our conclusive results, the heterogeneity of the data was high only in the analysis of the association between ppBMI and offspring's HOMA‐IR. This indicates the presence of other necessary determining factors in the background.

Despite the limitations, our meta‐analysis demonstrates positive associations between maternal overnutrition and offspring's IR and highlights the determining role of both ppBMI and excessive GWG (independent of ppBMI) on offspring's health. With regard to the still rising prevalence of obesity/overweight in reproductive‐aged women, further effective preventive strategies are needed such as bariatric surgery before pregnancy or nutritional corrections in reproductive‐aged women even before conception and also during pregnancy. We have proven the determining role of offspring's actual BMI or other measures of adiposity in the association between ppBMI and offspring's IS. This finding emphasizes the importance of prevention of obesity especially in children of obese non‐diabetic mothers.

## CONFLICTS OF INTEREST

The authors declare that they have no conflicts of interest.

## CONTRIBUTIONS

PH is the project leader. EP and SE formulated the research question. ZS, MB, BE, MS and AG contributed to the study design. SE and EK conducted the study selection, data extraction and the risk of bias assessment. EP resolved the disagreements. DN, ZS, MB, BE, MS and AG analysed and interpreted the data. EP and SE wrote the paper. All authors contributed to critically revising the article and gave approval of the final manuscript.

## APPENDIX A SEARCH STRATEGY

The review was conducted by searching MEDLINE (via PubMed), EMBASE, Scopus and CENTRAL databases until April 22, 2019.

Search query:

(child* OR offspring OR adolescen* OR son OR daughter OR adult*) AND (maternal OR gestation* OR pregestat* OR pre‐gestat* OR prenat* OR perinat* OR pregnan* OR prepregnan* OR pre‐pregnan* OR preconception* OR pre‐conception* OR periconception* OR peri‐conception* OR pregravid* OR pre‐gravid* OR antenatal* OR mother) AND (obesity OR overnutrit* OR over‐nutrit* OR overweight OR adiposity OR obese OR ‘body weight’ OR ‘weight gain’ OR ‘weight change’ OR ‘weight growth’ OR ‘body mass’ OR ‘body mass index’ OR BMI OR ‘skinfold thickness’) AND (insulin OR hyperinsulin* OR hyperinzulin* OR glucose) with restrictions to English language and human studies.

The symbol ‘*’ represents truncation.

All search results were combined in and duplicates were removed with a reference manager software, EndNote X7.0.2 (Clarivate Analytics, Philadelphia, PA, USA).

We manually searched the reference lists of eligible articles and relevant reviews to identify additional studies. Citation searches for all studies that met the inclusion criteria and all related systematic reviews were performed using Google Scholar Citations.

No supplementary information was obtained from investigators of the original clinical studies; only published data were used.

## APPENDIX B PRISMA 2009 CHECKLIST

**Table:**

Section/topic	#	Checklist item	Reported on page #
**TITLE**			
Title	1	Identify the report as a systematic review, meta‐analysis or both.	1
**ABSTRACT**			
Structured summary	2	Provide a structured summary including, as applicable: Background; objectives; data sources; study eligibility criteria, participants and interventions; study appraisal and synthesis methods; results; limitations; conclusions and implications of key findings; systematic review registration number.	1
**INTRODUCTION**			
Rationale	3	Describe the rationale for the review in the context of what is already known.	3–4
Objectives	4	Provide an explicit statement of questions being addressed with reference to participants, interventions, comparisons, outcomes and study design (PICOS).	4
**METHODS**			
Protocol and registration	5	Indicate if a review protocol exists, if and where it can be accessed (e.g., web address), and, if available, provide registration information including registration number.	4
Eligibility criteria	6	Specify study characteristics (e.g., PICOS, length of follow‐up) and report characteristics (e.g., years considered, language, publication status) used as criteria for eligibility, giving rationale.	4–5, 29
Information sources	7	Describe all information sources (e.g., databases with dates of coverage, contact with study authors to identify additional studies) in the search and date last searched.	4, Appendix A
Search	8	Present full electronic search strategy for at least one database, including any limits used, such that it could be repeated.	Appendix A
Study selection	9	State the process for selecting studies (i.e., screening, eligibility, included in systematic review and, if applicable, included in the meta‐analysis).	4–5
Data collection process	10	Describe method of data extraction from reports (e.g., piloted forms, independently, in duplicate) and any processes for obtaining and confirming data from investigators.	4, Appendix A
Data items	11	List and define all variables for which data were sought (e.g., PICOS, funding sources) and any assumptions and simplifications made.	5
Risk of bias in individual studies	12	Describe methods used for assessing risk of bias of individual studies (including specification of whether this was done at the study or outcome level), and how this information is to be used in any data synthesis.	5
Summary measures	13	State the principal summary measures (e.g., risk ratio, difference in means).	6–7
Synthesis of results	14	Describe the methods of handling data and combining results of studies, if done, including measures of consistency (e.g., *I* ^2^) for each meta‐analysis.	6–7
Risk of bias across studies	15	Specify any assessment of risk of bias that may affect the cumulative evidence (e.g., publication bias, selective reporting within studies).	5
Additional analyses	16	Describe methods of additional analyses (e.g., sensitivity or subgroup analyses, meta‐regression), if done, indicating which were pre‐specified.	6
**RESULTS**			
Study selection	17	Give numbers of studies screened, assessed for eligibility and included in the review, with reasons for exclusions at each stage, ideally with a flow diagram.	7, Figure 1
Study characteristics	18	For each study, present characteristics for which data were extracted (e.g., study size, PICOS, follow‐up period) and provide the citations.	7–9, 29
Risk of bias within studies	19	Present data on risk of bias of each study and, if available, any outcome level assessment (see Item 12).	9, Appendix E
Results of individual studies	20	For all outcomes considered (benefits or harms), present, for each study: (a) simple summary data for each intervention group (b) effect estimates and confidence intervals, ideally with a forest plot.	10–12, Figures 2, 3, 4, 5
Synthesis of results	21	Present results of each meta‐analysis done, including confidence intervals and measures of consistency.	10–12, Figures 2, 3, 4, 5
Risk of bias across studies	22	Present results of any assessment of risk of bias across studies (see Item 15).	12
Additional analysis	23	Give results of additional analyses, if done (e.g., sensitivity or subgroup analyses, meta‐regression [see Item 16]).	10–12
**DISCUSSION**			
Summary of evidence	24	Summarize the main findings including the strength of evidence for each main outcome; consider their relevance to key groups (e.g., healthcare providers, users and policy makers).	13–17
Limitations	25	Discuss limitations at study and outcome level (e.g., risk of bias), and at review level (e.g., incomplete retrieval of identified research, reporting bias).	18
Conclusions	26	Provide a general interpretation of the results in the context of other evidence, and implications for future research.	2, 18
**FUNDING**			
Funding	27	Describe sources of funding for the systematic review and other support (e.g., supply of data); role of funders for the systematic review.	Title page

From: Moher D, Liberati A, Tetzlaff J, Altman DG, The PRISMA Group (2009). Preferred Reporting Items for Systematic Reviews and Meta‐analyses: The PRISMA Statement. PLoS Med 6(7): e1000097. doi: 10.1371/journal.pmed1000097.

For more information, visit: www.prisma‐statement.org.

## APPENDIX C CHARACTERISTICS OF THE EXCLUDED STUDIES

**Table:**

Author, year, country	Study design	Maternal characteristics			Offspring			Reason of exclusion
		Age (year)	Sample size	Assessment	Age (year)	Outcome		
						Unadjusted for offspring's anthropometry	Adjusted for offspring's anthropometry	
Bucci et al., 2016, Finland	Retrospective cohort	22–34 (range)	17	Maternal groups based on BMI prior to delivery: ≥28.1 vs. ≤26.3	71.5 ± 0.9 (mean ± SD)	Higher level of: Insulin glucose muscle insulin sensitivity^*^	—	BMI assessed only prior to delivery
Eriksson et al., 2014, The Netherlands	Retrospective cohort	22–34 (range)	13,345	Maternal groups based on BMI prior to delivery: ≥28 vs. ≤24	56–70 (range)	Higher OR for diagnosed diabetes mellitus Type 2^*^	‐	BMI assessed only prior to delivery
Mi et al., 2000, China	Prospective cohort	22–33 (range)	627	BMI as continuous variable	43–46 (range)	—	Negative regression coefficient for glucose insulin^*^	BMI assessed only at 15th and 38th weeks of gestation
Shaikh et al., 2015, Pakistan	Cross‐sectional	25.23 ± 3.68 (mean ± SD)	24	Maternal groups based on BMI in the third trimester: <22 vs. >23	Newborn	Higher level of insulin glucose^*^ HOMA‐IR	—	BMI assessed between 28 and 40 weeks of gestation
Veena et al., 2013, India	Prospective cohort	23.5 ± 4.0 (mean ± SD)	504	BMI at 30th week of gestation as continuous variable	9.5	Positive sdr regression coefficient for insulin^*^ glucose	Positive sdr regression coefficient for insulin HOMA‐IR negative regression coefficient for glucose	BMI assessed only at 30th week of gestation

Abbreviations: BMI, body mass index; HOMA‐IR, Homeostatic Measurement Assessment for Insulin Resistance; IQR, interquartile range; SD, standard deviation; sdr, standardized.

^*^ *p* < 0.05.

## APPENDIX D ADJUSTMENTS WITH COVARIATES OF THE INCLUDED STUDIES

**Table:**

Author, year, country	Adjustments with covariates
Brandt, 2014, Germany	Offspring: Gender.
Dello Russo et al., 2013, Germany, Hungary, Italy, Cyprus, Spain, Estonia, Sweden, Belgium	Mothers: Age, BMI, gestational age, alcohol, and smoking during pregnancy, gestational diabetes, hypertension. Offspring: Sex, age, practice of sport, breastfeeding duration.
Derraik et al., 2015, New Zealand	Mothers: Birth order, age. Offspring: Age, sex, birth weight.
Gaillard et al., 2014, The Netherlands	Mothers: ppBMI, age, educational level, ethnicity, parity, gestational age, height, smoking, alcohol, calorie intake during pregnancy, folic acid supplement use, delivery mode. Offspring: Sex, age, breastfeeding duration, age at introduction of solid foods, duration of TV‐watching, with/without BMI.
Gaillard et al., 2016, Australia	Mothers: ppBMI, age, ethnicity, educational level, household income, parity, smoking during pregnancy, total GWG, hypertension, gestational diabetes, caesarean delivery, gestational age. Offspring: Sex, age, birth weight, and length, breastfeeding duration, infant length, and weight growth, paternal BMI, Tanner stage, smoking, alcohol, calorie intake, physical activity, with/without BMI.
Hochner et al., 2012, Israel	Mothers: Age, parity, smoking, socioeconomic status, years of education, medical condition, gestational week Offspring: Ethnicity, sex, birth weight, smoking, physical activity, years of education, with/without BMI
Hrolfsdottir et al., 2015, Denmark	Mothers: ppBMI, age, parity, smoking, educational level. Offspring: Sex, whether offspring think their father is overweight.
Jeffery et al., 2006, United Kingdom	Unadjusted.
Maftei et al., 2015, Australia	Mothers: Gestational diabetes. Offspring: With/without z‐BMI.
Mingrone et al., 2008, Italy	Unadjusted.
Oostvogels et al., 2014, The Netherlands	Mothers: Age, height, education level, ethnicity, parity, hypertension, smoking during pregnancy, gestational age. Offspring: Sex, birth weight, breastfeeding duration, screen time per day, energy intake, with/without weight‐for‐length gain.
Perng et al., 2014, USA	Mothers: ppBMI, race/ethnicity, parity, smoking, household income. Offspring: Sex, age, father's BMI, with/without DXA total fat mass index.
Sauder et al., 2017, USA	Offspring: Sex, race/ethnicity, age, Tanner stage, age by Tanner interaction, with/without BMI**.**
Tam et al., 2018, China	Mothers: ppBMI, insulin sensitivity, parity, age, gestational age. Offspring: Sex, age, breastfeeding duration, exercise level.
Winham et al., 2006, USA	Unadjusted.

Abbreviations: DXA, dual‐energy X‐ray absorptiometry; GWG, gestational weight gain; ppBMI, prepregnancy BMI.

## APPENDIX E QUALITY IN PROGNOSIS STUDIES (QUIPS) TOOL (ADAPTED FOR THE STUDY)

**Table:**

Modified QUIPS items	Brandt 2014	Dello Russo 2013	Derraik 2015	Gaillard 2014	Gaillard 2016	Hochner 2012	Hrolfsdottir 2015	Jeffery 2006	Maftei 2015	Mingrone 2008	Oostvogels 2014	Perng 014	Sauder 2017	Tam 2018	Winham 2006
1. Study participation															
Source of target population adequately described for key characteristics including age, gender, pregnancy characteristics	Yes	Yes	Yes	Yes	Yes	Yes	Yes	Yes	Yes	Yes	Yes	Yes	Yes	Yes	Yes
The method used to identify the study population is described adequately	Yes	Yes	Yes	Yes	Yes	Yes	Yes	Yes	Yes	Yes	Yes	Yes	Yes	Yes	Yes
Recruitment period is stated	Yes	Yes	Yes	Yes	Yes	Yes	Yes	Yes	Yes	Yes	Yes	Yes	Yes	Yes	No
Place of recruitment (setting and location) is stated	Yes	Yes	Yes	Yes	Yes	Yes	Yes	Yes	Yes	Yes	Yes	Yes	Yes	Yes	Yes
Inclusion and exclusion criteria are clearly defined	Yes	Yes	Yes	Yes	Yes	Yes	Yes	Yes	Yes	Yes	Yes	Yes	Yes	Yes	No
There is adequate participation in the study by eligible individuals >80%^a^	No	NA	NA	No	No	No	No	Yes	No	NA	No	No	No	No	NA
Baseline characteristics of the participants are collected including gestational age, birth weight, age, gender	Yes	Yes	Yes	Yes	Yes	Yes	Yes	Yes	Yes	Yes	Yes	Yes	Yes	Yes	Yes
Summary: Study participation	Low	Low	Low	Low	Low	Low	Low	Low	Low	Low	Low	Low	Low	Low	Moderate
2. Study attrition															
All the included patients have outcome data, or the dropout rate is maximum 20%^a^	No	NA	NA	No	No	No	No	Yes	No	NA	No	No	No	No	NA
Attempts to collect information on participants who dropped out^a^	Yes	NA	NA	Yes	Yes	Yes	Yes	Yes	Yes	NA	Yes	Yes	Yes	Yes	NA
Reasons for lost to follow‐up are described^a^	Yes	NA	NA	Yes	Yes	Yes	Yes	Yes	Yes	NA	Yes	Yes	Yes	Yes	NA
Key characteristics (age, gender, pregnancy characteristics) information on those lost to follow‐up^a^	Yes	NA	NA	Yes	Yes	Yes	Yes	Yes	Yes	NA	Yes	Yes	Yes	Yes	NA
There are no important differences between key characteristics and outcomes in participants who completed the study and those who did not^a^	Unclear	NA	NA	Unclear	Unclear	Yes	Unclear	Unclear	Unclear	NA	Unclear	Unclear	Unclear	Unclear	NA
Summary: Study attrition	Moderate	NA	NA	Moderate	Moderate	Low	Moderate	Low	Moderate	NA	Moderate	Moderate	Moderate	Moderate	NA
3. Prognostic factor measurement															
Definition of maternal nutritional state (ppBMI and/or GWG) and the method of measurements are clearly described	Yes	Yes	Yes	Yes	Yes	Yes	Yes	Yes	Yes	Yes	Yes	Yes	Yes	Yes	Yes
Method of measuring of maternal nutritional state is valid and reliable^b^	Yes	Partly	Partly	Partly	Partly	Partly	Yes	Partly	Partly	Partly	Partly	Partly	Yes	Yes	Yes
Continuous variables (ppBMI and/or GWG) are reported or appropriate cut points are used (GWG categories in accordance with the IOM recommendations)	Yes	Yes	Yes	Yes	Yes	Yes	Yes	Yes	Yes	Yes	Yes	Yes	Yes	Yes	Yes
Method and setting of measuring maternal nutritional state are the same for all study participants	Yes	Yes	Yes	Yes	Yes	Yes	Yes	Yes	Yes	Yes	Yes	Yes	Yes	Yes	Yes
All the included participants have complete data for the maternal nutritional state	Yes	Yes	Yes	Yes	Yes	Yes	Yes	Yes	Yes	Yes	Yes	Yes	Yes	Yes	Yes
Appropriate method was used to replace the missing data on maternal nutritional state^c^	NA	NA	NA	NA	NA	NA	NA	NA	NA	NA	NA	NA	NA	NA	NA
Summary: Measurement of the maternal nutritional state	Low	Low	Low	Low	Low	Low	Low	Low	Low	Low	Low	Low	Low	Low	Low
4. Outcome measurement															
Definition of outcome: Fasting insulin level	Yes	Yes	Yes	Yes	Yes	Yes	Yes	NA	NA	Yes	NA	NA	Yes	Yes	Yes
Definition of outcome: HOMA‐IR	NA	Yes	NA	NA	Yes	NA	Yes	Yes	Yes	NA	NA	Yes	Yes	Yes	NA
Definition of outcome: Fasting glucose level	NA	NA	NA	NA	Yes	Yes	Yes	NA	NA	NA	Yes	NA	NA	Yes	NA
Valid and reliable measurement of outcome	Yes	Yes	Yes	Yes	Yes	Yes	Yes	Yes	Yes	Yes	Yes	Yes	Yes	Yes	Yes
Method and setting of outcome measurement is the same for all study participants	Yes	Yes	Yes	Yes	Yes	Yes	Yes	Yes	Yes	Yes	Yes	Yes	Yes	Yes	Yes
Summary: Outcome measurement	Low	Low	Low	Low	Low	Low	Low	Low	Low	Low	Low	Low	Low	Low	Low
5. Study confounding															
Maternal age, as an important confounder is measured	Yes	Yes	Yes	Yes	Yes	Yes	Yes	Yes	Yes	Yes	Yes	Yes	Yes	Yes	Yes
Maternal smoking habits during pregnancy, as an important confounder is measured	Yes	Yes	Yes	Yes	Yes	Yes	Yes	No	Yes	No	Yes	Yes	Yes	Yes	No
Maternal socioeconomic status/maternal educational level, as an important confounder is measured	Yes	Yes	No	Yes	Yes	Yes	Yes	Yes	Yes	No	Yes	Yes	No	No	No
Birth weight, as an important confounder is measured	Yes	Yes	Yes	Yes	Yes	Yes	Yes	Yes	No	Yes	Yes	Yes	Yes	No	Yes
Breastfeeding, as an important confounder is measured	No	Yes	No	Yes	Yes	No	No	No	No	No	Yes	Yes	No	Yes	No
Offspring's age, as an important confounder is measured	Yes	Yes	Yes	Yes	Yes	Yes	Yes	Yes	Yes	Yes	Yes	Yes	Yes	Yes	Yes
Offspring's adiposity (weight or BMI or fat mass index), as an important confounder is measured	Yes	Yes	Yes	Yes	Yes	Yes	Yes	Yes	Yes	Yes	Yes	Yes	Yes	Yes	No
The measured confounding factors are clearly defined	Yes	Yes	Yes	Yes	Yes	Yes	Yes	Yes	Yes	Yes	Yes	Yes	Yes	Yes	Yes
Valid and reliable measurement of confounders	Yes	Yes	Yes	Yes	Yes	Yes	Yes	Yes	Yes	Yes	Yes	Yes	Yes	Yes	Yes
Method and setting of confounding measurement are the same for all study participants	Yes	Yes	Yes	Yes	Yes	Yes	Yes	Yes	Yes	Yes	Yes	Yes	Yes	Yes	Yes
An appropriate method was used for replacing missing confounder data (e.g., imputation)	NA	NA	NA	NA	NA	NA	NA	NA	NA	NA	NA	NA	NA	NA	NA
Important confounders are accounted for in the study design (e.g., initial assembly of comparable groups or matching)	Partly	Yes	Partly	Yes	Yes	Partly	Partly	Partly	No	No	Yes	Yes	Partly	Partly	No
Confounders considered important by the authors of the article, are accounted for in the analysis (e.g., appropriate adjustment, stratification, multivariate regression)	No	Yes	No	Yes	Yes	Partly	No	No	No	No	Yes	Partly	No	Partly	No
Summary: Study confounding	High	Low	High	Low	Low	Moderate	High	High	High	High	Low	Moderate	High	High	High
6. Statistical analysis and reporting															
The analytical strategy is described in detail, the adequacy of the analysis can be judged	Yes	Yes	Yes	Yes	Yes	Yes	Yes	Yes	Yes	Yes	Yes	Yes	Yes	Yes	Yes
Strategy for model building is appropriate and is based on a conceptual framework or model	Yes	Yes	No	Yes	Yes	Yes	Yes	Yes	Yes	Yes	Yes	Yes	Yes	Yes	Yes
The results are reported in detail, no selective reporting can be observed	Yes	Yes	Unclear	Yes	Yes	Yes	Unclear	Yes	Unclear	Unclear	Yes	Yes	Yes	Yes	Unclear
Summary: Statistical analysis and presentation	Low	Low	Moderate	Low	Low	Low	Low	Low	Low	Low	Low	Low	Low	Low	Low

Ratings: Items are rated with yes (low risk of bias), unclear (unknown risk of bias), partly (moderate risk of bias) or no (high risk of bias).

For the 1st–4th and the 6th domains: If two of the items were rated as unclear, partly or no, then the specific main domain was considered to carry moderate risk of bias. If three or more of the items were rated as unclear, partly or no, then the specific main domain was considered to be carrying high risk of bias. Because the reasons for lost to follow‐up (dropouts) were described in all prospective studies and they randomly affected the participants, the dropout rate higher than 20% was unlikely to cause selection bias (see domains 1st and 2nd).

For the 5th domain: If maximum one of the items was rated as no plus one or two were rated as partly, then the specific main domain was considered to be carrying moderate risk of bias. If two or more of the items was rated as no, then the specific main domain was considered to be carrying high risk of bias.

Abbreviations: BMI, body mass index; GWG, gestational weight gain; HOMA‐IR, Homeostatic Measurement Assessment for Insulin Resistance; IOM, Institute of Medicine; NA, not applicable due to study design; ppBMI, prepregnancy BMI.

^a^ Items only applicable in case of prospective studies. Retrospective ones were not assessed.

^b^ In case of self‐reported maternal data the item was rated as partly. If data were measured or obtained from records, the item was rated as yes.

^c^ Only those patients were included in the studies, who had appropriate data on maternal nutritional state. Therefore, this item was not assessed.

## APPENDIX F BETA REGRESSION COEFFICIENTS OF THE ASSOCIATION BETWEEN ppBMI and OFFSPRING'S INSULIN LEVEL

Beta regression coefficients describing the association between prepregnancy body mass index (BMI) and offspring's insulin level without adjustment for offspring's birthweight and actual BMI (*I* ^2^ = 80.06%, *p* = 0.007). Black squares show beta values with the area reflecting the weight assigned to the individual studies. Horizontal bars indicate 95% confidence intervals. Diamonds show the overall point estimate with 95% confidence intervals
